# Prevalence of hearing loss at primary health care clinics in South Africa

**DOI:** 10.4314/ahs.v18i2.16

**Published:** 2018-06

**Authors:** Christine Louw, De Wet Swanepoel, Robert H Eikelboom, Jannie Hugo

**Affiliations:** 1 Department of Speech-Language Pathology and Audiology, University of Pretoria, Pretoria, South Africa; 2 Ear Science Institute Australia, Subiaco, Australia; 3 Ear Sciences Centre, The University of Western Australia, Nedlands, Australia; 4 Department of Family Medicine, University of Pretoria, Pretoria , South Africa

**Keywords:** Hearing loss, primary health care clinics, South Africa

## Abstract

**Background:**

Hearing loss prevalence data in South Africa is scarce, especially within primary health care settings.

**Objectives:**

To determine; (i) the prevalence of hearing disorders in patients ≥3 years of age attending two primary health care clinics, and (ii) the nature and characteristics of hearing disorders at these primary health care clinics.

**Method:**

A cross-sectional design was used at two primary health care clinics. Non-probability purposive sampling was used to screen participants at clinics for hearing loss with pure tone audiometry. A total of 1236 participants were screened (mean age 37.8 ±17.9 years). Diagnostic testing was available for confirmation of hearing loss on participants who failed the screening.

**Results:**

Hearing loss prevalence was 17.5% across both clinics. Most hearing losses were bilateral (70.0%) and were of a sensorineural nature (84.2%).

**Conclusion:**

Hearing loss prevalence was comparable at both primary health care clinics. Participants 40 years and older were at significantly higher risk for hearing loss. The current study is the first attempt to establish hearing loss prevalence for primary health care clinics in South Africa.

## Introduction

Hearing loss is a major public health concern affecting more than 1.33 billion people globally in 2015^1^. As one of the leading contributors to the global burden of disease, it currently ranks fifth on the global causes of years lived with disability index, higher than other chronic diseases such as diabetes or dementia^1^. A combination of factors is responsible for the upward trend in the global hearing loss epidemic. These include increased life expectancy leading to the number one cause of hearing loss, aging. The widespread use of ototoxic treatments for diseases such as cancer and tuberculosis, and occupational and recreational noise exposure without appropriate protection are other major contributors to the global burden of hearing loss^2^.

Hearing loss has a devastating effect on the individual with some of the resulting sequelae including academic failure, higher unemployment, poorer general health, social isolation and an increased incidence of depression^3^,^4^. In addition to its individual effects, hearing loss puts an immense financial burden on society. Health care costs, excluding rehabilitation services such as hearing devices and cochlear implants, are estimated to be in the range of US67 -107 billion annually^5^. The financial burden of unaddressed hearing loss, however, is far worse for developing countries that are challenged by pre-existing poverty, environmental risk factors and life-threatening diseases^5^. Sub-Saharan Africa is one of the developing world regions with substantially higher hearing loss prevalence compared to developed countries^3^,^6^. Available reports indicate an estimated prevalence of 11.5 to 20.3% for adults (≥ 15 years) and 1.2 to 3.0% for children (5 – 14 years) in SSA compared to 4.0 to 6.4% (adults ≥ 15 years) and 0.3 to 0.6% (children 5 – 14 years) in high-income countries^6^. Hearing loss prevalence in sub-Saharan Africa may however be underestimated as population-based studies are limited^7^.

Apart from a preliminary population-based study conducted in the Cape Town metropolitan area^8^, limited hearing loss data is available also in South Africa. Similarly hearing loss prevalence in South African communities and those attending primary health care (PHC) facilities is unknown. It is an important priority to obtain local data for hearing loss prevalence at PHC level where communicable (i.e. tuberculosis) and non-communicable diseases (i.e. diabetes), which are associated with acquired hearing losses, are being treated^9^,^10^. Considering that an estimated 50% of the burden of hearing loss could be prevented^11^, implementing hearing care services such as prevention, management and intervention are needed at PHC level. Determining the prevalence of hearing loss is required for adequate health planning to increase access to hearing care services within communities. This study describes the prevalence and nature of hearing loss of those attending two South African PHC clinics.

## Materials and methods

This research project was approved by the Institutional Research Board of the University of Pretoria, South Africa and was part of a larger community oriented primary care (COPC) project in Gauteng province in the City of Tshwane^12^.

### Selection and description of participants

A cross-sectional design was used at two PHC clinics (PHC clinic 1 and PHC clinic 2) situated in different underserved communities in Tshwane. Non-probability purposive sampling was used to screen participants as it was a clinical, non-experimental set-up and results would therefore be representative of the clinic population. At PHC clinic 1, universal screening took place by offering all individuals who visited the clinic a hearing screening free of charge. At PHC clinic 2, all individuals who were available during the time that the services were delivered and who wanted their hearing tested were screened free of charge. Only individuals visiting the clinic as patients were selected as participants. Diagnostic testing was available for confirmation of hearing loss on participants who failed the screening. Participants aged three years and older were included in the study. This criterion was included as the preferred method of testing children younger than 3 years of age is visual response audiometry (VRA) which was not available at the clinics. Only participants who provided signed consent (children had to provide assent along with a signed consent letter from their parent/caregiver) and who completed the screening protocol (i.e. completed a re-screen upon referral of initial screen) were included in the study. Instructions were provided in English or Afrikaans. Written instructions in Sepedi were used if participants did not understand English or Afrikaans. If participants were unable to understand one of these three languages, a health care nurse who was available at the specific time, was asked to translate the information. Participants who presented with a mixed or conductive hearing loss were referred to the clinics' general practitioner for further medical examination and intervention. Participants who presented with a sensorineural hearing loss (SNHL) were referred to the nearest district hospital for a hearing aid fitting evaluation.

### Procedures

#### Hearing screening

Hearing screening was facilitated by undergraduate audiology students from the University of Pretoria under supervision of an experienced audiologist (first author). Otoscopy was performed with a handheld Welch Allyn otoscope (Welch Allyn, South Africa) as a pre-screen to determine any obvious abnormalities of the external ear canal or tympanic membrane. Any obvious abnormalities of the external ear canal or tympanic membrane were noted. Testing was conducted in an examination room without sound isolation. Due to time and facility constraints at clinics more than one participant was examined at the same time in a room in some instances. In these instances, more than one student audiologist was available to evaluate participants. Smartphone hearing screening was performed using two sets of Samsung Galaxy Pocket Plus S5301 phones running the validated hearScreen™ Android OS application with calibrated supra-aural Sennheiser HD202 II headphones (Sennheiser, Wedemark, Germany)^13^,^14^. The application's integrated real-time monitoring of ambient noise levels provided a measure of quality control^13^. Screening audiometry was conducted, according to recommended guidelines^15^ using a 1, 2 and 4 kHz sweep with screening levels of 25 dB HL or 35 dB HL for participants younger than 15 years and 15 years and older respectively^13^. These age cut-offs are in line with the World Health Organization^16^. Immediately following a fail result, the participant was re-screened. Only those who failed both screenings were considered to have failed the hearing screening test. Findings from previous studies indicate that hearScreen™ can be used as a reliable screening tool, also in PHC settings^13^,^17^. To determine screening specificity in this particular study, diagnostic testing was performed on a group of 111 participants who passed the screening.

#### Diagnostic testing

To determine the prevalence and nature of hearing loss, pure tone audiometry was performed on the same day on participants who failed the screening for a second consecutive time. Automated pure tone audiometry (air- and bone conduction) was performed at 0.5, 1, 2, 4 kHz using a Type 2 Clinical Audiometer (KUDUwave, eMoyo, South Africa). Insert earphones were placed deep in the ear canal with circumaural headphones placed over the ears to improve attenuation of ambient noise, and to minimize the occlusion effect. An automated threshold-seeking paradigm was utilized with a similar threshold-seeking method used in manual test configuration i.e. the modified Hughson-Westlake method. Air and bone conduction thresholds were determined with masking of the non-test ear when indicated. The software actively monitored ambient noise levels across octave bands throughout the test procedures in both clinics. Whenever the noise exceeded the maximum ambient noise level allowed for establishing a threshold, the test operator waited for the transient noise to subside.

#### Data analysis

Diagnostic testing confirming a hearing loss informed the prevalence rate for this population. The presence of a hearing loss was defined as a pure tone threshold average (0.5, 1, 2 or 4 kHz) greater than 25 dB HL in one or both ears^18^. A hearing loss was classified as conductive when the average difference between the pure tone air conduction and bone conduction thresholds (0.5 kHz – 4 kHz) was 15 dB HL or greater with normal air conduction thresholds^18^. A hearing loss was classified as a sensorineural hearing loss (SNHL) when the pure tone air and bone conduction thresholds (0.5 kHz – 4 kHz) were abnormal (> 25 dB HL) with an average air-bone gap less than 15 dB HL. The classification of a mixed hearing loss (conductive and sensorineural) entailed abnormal air and bone conduction thresholds with an average air-bone gap of 15 dB HL or greater. Another hearing loss category (“other”) was added in the current study to include participants with different types of hearing losses in the two ears i.e. a SNHL and conductive hearing loss. The degree of the hearing loss was classified as mild (> 25 dB HL and ≤ 40 dB HL), moderate (> 40 dB HL and ≤ 55 dB HL), moderate to severe (> 55 dB HL and ≤ 70 dB HL) and severe to profound (> 70 dB HL)^18^. A unilateral hearing loss was obtained when one ear had normal hearing with a hearing loss in the other ear. A bilateral hearing loss indicated a hearing loss present in both ears. Data analysis was performed using SPSS v23 (Armonk, New York). Demographic data, screening and diagnostic results were analysed and presented using descriptive statistics. A one way ANOVA analysis was performed to evaluate the effect of age, gender and race on the presence of a hearing loss in the sample, with p < .05 indicating a significant association.

## Results

A total of 1236 participants were included in the study (PHC 1: n=633; PHC: n=603) (Table 1). The mean age was 37.8 years (±17.9 years, range 3 – 97 years). Twenty six participants (22 adults, four children) at PHC clinic 1 and two participants (2 adults) at PHC clinic 2 were excluded from the study because the screening protocol was not completed due to operator error. Two other participants were omitted from the study group at PHC clinic 2 because their date of birth was not captured. Two hundred and sixteen (17.5%) participants failed the hearing screening (PHC 1 = 18.8%, PHC 2 = 16.1%). 4.8% participants failed in the 3–14 years category, whilst 10.5% and 25.4% failed in the 15 – 39 years and > 40 years categories respectively. Of the 216 participants who failed, 138 participants were tested diagnostically whilst 78 did not attend the diagnostic assessment.

**Table 1:** Demographic categories across the study population (n=1236)

Characteristics	Combined sample (n=1236)	PHC 1 (n=633)	PHC 2 (n=603)
**Race**			
*Black*	64.4% (796)	100.0% (633)	27.0% (163)
*White*	35.6% (440)	−	73.0% (440)
**Gender**			
*Male*	28.7% (355)	25.3% (160)	32.3% (195)
*Female*	71.3% (881)	74.7% (473)	67.7% (408)
**Age**			
*3 – 14 years*	10.2% (126)	3.3% (21)	17.4% (105)
*15 – 39 years*	45.5% (562)	52.8% (334)	37.8% (228)
*≥40 years*	44.3% (548)	43.1% (278)	44.7% (270)

One hundred and twenty (9.7%) of the participants (mean age 49.8±19.8 years) presented with a confirmed hearing loss (PHC 1=9.3%; PHC 2=10.1%) (Table 2). When the 78 participants who failed the screening, but who did not attend for diagnostic testing, are included, the prevalence is 17.5% across both clinics. The majority of persons with hearing loss presented with a bilateral loss (70.0%, n=84). SNHL (uni- and bilateral) was the most common type of hearing loss (84.2%, n=101) followed by conductive (3.3%, n=4) and mixed hearing loss (1.7%, n=2). Sixty seven adults and two children presented with a bilateral sensorineural hearing loss (Table 2).

**Table 2:** Nature of hearing loss in adults and children (n=120)

	Conductive hearing loss	Mixed hearing loss	SNHL	Other (SNHL & mixed, SNHL & conductive, conductive & mixed)
*≥ 15 years*	1.7% (2)	1.7% (2)	81.0% (97)	9.2% (11)
*3–14 years*	1.7% (2)	−	3.3% (4)	1.7% (2)

Thirteen participants (10.8%) were diagnosed with combinations of SNHL, conductive or mixed losses in respective ears (Table 3). Nineteen participants (9 participants with conductive hearing loss, 10 participants with mixed hearing loss) were referred for further medical investigation. The majority of the hearing impaired (38.0%) participants presented with a moderate degree of hearing loss (Table 4). Race and gender did not have a significant effect on hearing loss (p>.05; ANOVA) but age had a significant effect (p< .05) with hearing sensitivity decreasing by 3.4 dB (95% CI: 0.25 – 0.45) for every additional year.

**Table 3:** Prevalence and nature of hearing loss (n=120)

	Total (n=1236)	PHC 1 (n=633)	PHC 2 (n=603)
**All**	9.7% (120)	9.3% (59)	10.1% (61)
*3–14 years*	4.8% (6)	9.5% (2)	3.8% (4)
*15 – 39 years*	5.7% (32)	6.0% (20)	5.3% (12)
*≥40 years*	15.0% (82)	13.3% (37)	16.7% (45)
**Unilateral**			
*SNHL*	2.6% (32)	3.8.% (24)	1.3% (8)
*Conductive*	0.2% (2)	0.2% (1)	0.2% (1)
*Mixed*	0.2% (2)	−	0.3% (2)
**Bilateral**			
*SNHL*	5.6% (69)	4.1% (26)	7.1% (43)
*Conductive*	0.2% (2)	0.3% (2)	−
*Mixed*	−	−	−
**Other**			
*SNHL & Mixed*	0.6% (7)	0.3% (2)	0.8% (5)
*SNHL & Conductive*	0.4% (5)	0.6% (4)	0.1% (1)
*Conductive & Mixed*	0.1% (1)	−	0.1% (1)

**Table 4:** Degree of hearing loss (based on the worst ear pure tone average) (n=120)

Degree	Total (n=120)	PHC 1 (n=59)	PHC 2 (n=61)
**Mild**	29.2% (35)	32.2% (19)	26.2% (16)
**Moderate**	38.0% (45)	31.0% (18)	44.2% (27)
**Moderate to severe**	11.7% (14)	8.5% (5)	14.8.% (9)
**Severe to profound**	21.7% (26)	28.8% (17)	14.8% (9)

## Discussion

Hearing loss prevalence data in Africa varies greatly. The current study revealed a hearing loss prevalence of 17.5% at two PHC clinics in underserved communities in the Tshwane area. This is slightly higher than the 12.35% prevalence reported in the Cape Town metropolitan area^8^ whilst it is very similar to an estimated range of 11.4% –20.3% for sub-Saharan Africa^6^. Different contexts such as school settings or population-based contribute to the prevalence variation. The current study investigated hearing loss prevalence at PHC clinics. Different hearing test techniques employed also contribute to the variation. Also, in studies where pure tone audiometry was used as the screening method, there was also a wide variation in the intensity cut-off criteria i.e. 25 dB HL, 30 dB HL, 35 dB HL^7^. Using a stricter screen intensity such as 25 dB HL will identify milder hearing losses, and will produce a higher prevalence whilst a pure tone cut off at 40 bB HL will result in a lower prevalence as only moderate and severe losses will be included. The cut off criteria in the current study was of 25 dB HL (children) and 35 dB HL (adults). These intensities were selected to identify disabling hearing loss in children (>30 dB HL) and adults (>40 dB HL)^16^; however there may have been a small percentage of adults with slight hearing loss (> 25 dB < 35 dB) that may have passed the hearing screening.

There is an upward trend in hearing loss prevalence with increasing age^18^. The age differences in age groups also contribute to the prevalence discrepancy in Africa. Of the hearing loss participants was a significant factor in the current study with 15.0% of the participants 40 years or older presenting with a hearing loss Cruickshanks et al.^18^, showed that the risk of hearing loss, specifically SNHL increased by almost 90% for every five years of age. In the US, approximately two thirds of adults age 70 years and older present with a hearing loss^18^,^19^.

In the same age group in Europe at least 30% of men and 20% of women are affected by a hearing loss^20^. Only limited age-related prevalence evidence is available for sub-Saharan Africa. A study conducted in Nigeria indicated 6.1% of people aged 65 years and older self-reported hearing impairment^21^. Self-report of hearing loss is a quick and inexpensive way to determine hearing handicap, though the use of it in prevalence studies is limited if not validated against pure tone audiometry as the severity and site of lesion cannot be determined^22^,^23^. Two recent community based studies conducted in PHC settings also in the Tshwane area showed that increasing age had an impact on audiometric screening referral rate^24^,^17^. Both studies indicated that 25% of adults older than 40 years^17^ and 45 years^24^ were at risk hearing loss. In the current study, SNHL was the most common type of hearing loss observed among the hearing impaired participants who attended the diagnostic follow-up (n=120). Uni- and bilateral SNHL was observed in 2.6% and 5.6% of the participants respectively with 1.0% of the participants presenting with a bilateral loss which is sensorineural in nature in at least one ear. Conductive hearing loss, which refers to a middle ear pathology resulting in a loss of audibility, was observed in 0.2% of the hearing impaired participants with 0.5% of the hearing impaired presenting with a bilateral hearing loss which is conductive in nature in at least one ear.

A recent systematic review demonstrated that middle ear disease, that could ultimately lead to conductive hearing loss, is the most common cause not only in the schoolaged population, but also in the general population^7^. The screening cut off criteria was not aimed to identify mild conductive hearing losses, hence the low prevalence in the current study.

With the great variation in hearing loss prevalence due to different contexts, screening techniques and different age groups, it is imperative to recognize the lack of population-based studies in Africa. Hearing loss is a significant public concern being a highly prevalent and chronic condition. Individuals and communities are challenged by a variety of adverse effects as well as high costs. The negative effects and expenses in communities could be alleviated by implementing hearing care at PHC level. Implementing ear and hearing services at PHC level will require careful planning and identification of specific program goals and specification of care pathways. The current study is the first attempt in establishing hearing loss prevalence baselines and descriptions at PHC clinics. Novel hearing detection solutions, capitalizing on advances in technology and connectivity, which were used in the current study, provide the possibility to expand and decentralize hearing care services to PHC level.

## Study limitations

A limitation of the study was that participants younger than three years of age were not included. Furthermore, the population was sampled purposively and not randomly taking into consideration power and precision. This was mainly due to the clinical time and human resource constraints of the research setting.
